# Application of PCR-HRM method for microsatellite polymorphism genotyping in the *LDHA* gene of pigeons (*Columba livia*)

**DOI:** 10.1371/journal.pone.0256065

**Published:** 2021-08-19

**Authors:** Magdalena Jedrzejczak-Silicka, Adam Lepczynski, Filip Gołębiowski, Daniel Dolata, Andrzej Dybus

**Affiliations:** 1 Faculty of Biotechnology and Animal Husbandry, Laboratory of Molecular Biology, West Pomeranian University of Technology, Szczecin, Poland; 2 Department of Physiology, Cytobiology and Proteomics, West Pomeranian University of Technology, Szczecin, Poland; 3 QIAGEN Polska sp. z o.o., Wroclaw, Poland; 4 Bio-Techne sp. z o.o., Warszawa, Poland; 5 Faculty of Biotechnology and Animal Husbandry, Department of Genetics, West Pomeranian University of Technology, Szczecin, Poland; UCSI University, MALAYSIA

## Abstract

High-resolution melting (HRM) is a post-PCR method that allows to discriminate genotypes based on fluorescence changes during the melting phase. HRM is used to detect mutations or polymorphisms (e.g. microsatellites, SNPs, indels). Here, the (TTTAT)_3-5_ microsatellite polymorphism within intron 6 of the *LDHA* gene in pigeons was analysed using the HRM method. Individuals (123 homing pigeons) were genotyped using conventional PCR. Birds were classified into groups based on genotype type and the results were tested by qPCR-HRM and verified using sequencing. Based on the evaluated protocol, five genotypes were identified that vary in the number of TTTAT repeat units (3/3, 4/4, 3/4, 4/5, and 5/5). Sequencing have confirmed the results obtained with qPCR-HRM and verified that HRM is a suitable method for identification of three-allele microsatellite polymorphisms. It can be concluded that the high-resolution melting (HRM) method can be effectively used for rapid (one-step) discrimination of the (TTTAT)_3-5_ microsatellite polymorphism in the pigeon’s *LDHA* gene.

## Introduction

*LDHA–*lactate dehydrogenase isoform A (specific for muscle) is a member of a larger *LDH* gene family encoding L-lactate dehydrogenase (LDH, EC.1.1.1.27) [1,2]. Lactate dehydrogenase regulates aerobic and anaerobic metabolism, which has an significant impact on the physiological condition of the body, e.g. muscle endurance, recovery, aerobic capacity, survivability during racing competitions as well as overall physiological performance [1–4]. LDHA plays a crucial role as a coenzyme in the interconversion of pyruvate and lactate with nicotinamide dinucleotide [3,5,6]. The latest research suggest that lactate is a specific “cellular fuel”, including hippocampal nerve cells that are responsible for consolidating information from short-term to long-term memory, and spatial memory that determines navigation ability [3,7–9].

In 2002 for the first time the presence of *LDHA* gene polymorphism in pigeons was described [10]. Subsequent studies demonstrated that allele *A* of the *LDHA* gene (g.2582481G>A) was more frequent in the groups of pigeons with elevated homing performance [11,12]. The analysis of the effect of *LDHA* gene polymorphism (SNP, g.2582481G>A) on the racing performance of homing pigeons showed the difference in the value of ace points between animals with *GG* and *GA* genotypes. The study showed that allele *A* could be associated with better results of pigeons in competition flights [13,14]. Therefore, the *LDHA* gene is called the “Speed Gene” by commercial genotyping services. The (TTTAT)_3-5_ microsatellite polymorphism within intron 6 of the *LDHA* gene in pigeons has been described for the first time by Ramadan et al. [7] who found three different allele lengths (GenBank: AB744076, AB744077, AB744078) [3,7]. Ramadan et al. [3] found in Japanese and Egyptian pigeon populations that individuals carrying the *LDHA* S+ genotype (all genotypes containing minor allele S, i.e. 3 repeats, were classified as the S+) had higher EBV (estimated breeding value) resulting i.a. in greater survivability. Since Ramadan and co-workers [3] published their findings no other works has focused on that (TTTAT)_3-5_ microsatellite polymorphism in intron 6 of the *LDHA* gene.

Microsatellites (called simple sequence repeats − SSRs or short tandem repeats − STRs) are DNA segments composed of short (2–6 nucleotides), tandemly repeated (to over 200 times) motifs in the same orientation [15–18]. Short tandem repeats are often found in both intergenic and intragenic regions (rarely occurring within coding regions; trinucleotide repeats in or near genes are associated with certain inherited disorders) in prokaryotes and eukaryotes, including humans (accounting for approximately 3% of total human genome) [17–19]. The nature of microsatellites is complex—STRs are highly mutable (duplicative mutation rates range from 1 × 10^−3^ to 1 × 10^−4^ per generation) and demonstrate variability in length and sequence composition [17,18,20]. In contrast, other types of mutations (e.g., deletions, substitutions) are 4–5 times rarer with an estimated mutation rate of about 1.1 × 10^-n^ per site per generation [18,20]. The importance of microsatellite DNA as a powerful marker with a broad spectrum of applications should not be underestimated [21]. Microsatellites are widely used as valuable elements in population genetics because SSR markers are highly variable even cross closely related taxa [21]. Moreover, data obtained from microsatellite analyses have confirmed that individual microsatellites tend to be more polymorphic, and thus more informative, providing greater resolution in genetic studies than other molecular markers, including SNPs [21]. For example, Y-chromosome short tandem repeats (Y-STRs) are powerful human genome marker and have been used for many years in forensic practice [22]. The traditional approach used in microsatellites genotyping procedures is based on locus-specific polymerase chain reaction (PCR) for product amplification and polyacrylamide gel electrophoresis for genotypes identification [22]. Since the late 1990s, short tandem repeats (STRs) have been analysed using capillary electrophoresis (CE)–an automated technique utilizing laser-induced fluorescence to identify PCR products (amplicons are generated using fluorescence-labelled primers). However, studying microsatellites in the genome are more difficult compared to other common sequences.

Importantly, replication in microsatellite studies occurs during *in vitro* amplification of microsatellite sequences, resulting in the appearance of “stutter bands” or “shadow bands” in agarose electrophoresis or additional stutters picks in capillary electrophoresis [23–28]. “Stutter bands” may appear during the amplification of long and short tandem repeats [24,29–31]. The molecular mechanism of Taq DNA polymerase slippage during the amplification of repetitive DNA sequences and accumulation of products of different sizes (e.g. slipped strand mispairing; SSM) is not well explained [24,32]. Hommelsheim et al. [32] has found that polymerase DNA dissociates from the template because the appearance of hairpin loop structures (slippage). Due to the slippage process during the first PCR replication cycle [31], the obtained fragments act as megaprimers that randomly anneal with other sequences at variable positions (insertion or deletion of repeats may be occur). They are subsequently amplified by DNA polymerase, resulting in artifact formation that serve as templates in the subsequent amplification cycles, eventually including cause a ladder effect [24,32,33]. Slippage might take place at the active site of the enzyme or before substrate binding to the enzyme [34].

Moreover, besides of “stutter bands” there is another PCR artifacts class that must be taken into consideration–the heteroduplexes. As was stated by Kulibaba and Liashenko [35] amplification of microsatellite loci is usually accompanied by the formation of many additional fragments (artifacts) − heteroduplex DNA in the course of PCR is frequent. A heteroduplex is formed by the annealing of two individual single-stranded DNA in which antiparallel chains have different origin (derived from different alleles). In addition, mentioned artifacts are often situated near the target ones and cause difficulties in performing the precise genotyping [35]. On the other hand, sequencing allows to detect the stutter bands in PCR products, i.e. different product lengths due to an error in the number of repeat units [24].

We suppose that the reason for the lack of analysis of (TTTAT)_3-5_ microsatellite polymorphism in intron 6 of the *LDHA* gene polymorphism is caused by conventional PCR limitation. The main difficulty is the heteroduplexes formation during PCR reaction in case of heterozygotes samples and problems in the interpretation of the results of PCR products after agarose electrophoresis. Due to mentioned problems we applied the high resolution melting (HRM) method to detect the (TTTAT)_3-5_ microsatellite polymorphism in intron 6 of the *LDHA* gene as an alternative that offers easier results interpretation based on melting curves that directly translates into labour efficiency and reduction of time consumption. Based on the above information the aim of the study was to present a new approach to microsatellite polymorphism genotyping in the *LDHA* gene using HRM method with final results verification by DNA sequencing method.

## Methods

### Ethical approval

This study was carried out in strict accordance with the recommendations of the National Ethics Committee on Animal Experimentation. The protocol was approved by the Local Ethics Committee for Animal Testing of the West Pomeranian University of Technology in Szczecin (Protocol Number: 36/2012).

### DNA extraction

The present study comprised a total of 123 racing pigeons (from two racing lofts located in the Lubusz Province, Poland). Blood samples of all individuals were collected from the medial metatarsal vein into collection tubes containing anticoagulant (K_3_EDTA). Genomic DNA was extracted from 5 μl of whole peripheral blood using the MasterPure^TM^ DNA Purification Kit for Blood version II (Epicentre Biotechnologies, Madison, WI, USA), according to the vendor’s protocol.

### Genotyping with conventional PCR and gel electrophoresis

The (TTTAT)_3-5_ microsatellite polymorphism within intron 6 of the *LDHA* gene was analysed using conventional PCR reaction. PCR primers (**Table 1**) used in present study were designed based on the genomic sequence (GenBank accession no. NW_004973198.1, ref. 7) using Primer 3 software (https://primer3.ut.ee/). PCR reaction mixtures contained ~80 ng of genomic DNA, 15 pmol of each primer, 1 x PCR buffer, 1.5 mM MgCl_2_, 200 μM dNTP, 0.4 unit of *Taq* polymerase (Eurx, Gdańsk, Poland) and nuclease-free water (AppliChem, Darmstadt, German) in a total volume of 15 μl. The PCR amplification program was as follows: 5 min initial denaturation at 94°C followed by 35 cycles (denaturation at 94°C for 30 s, primer annealing at 61°C for 30 s and product synthesis at 72°C for 30 s) and final elongation at 72°C for 5 min (Biometra T-Personal thermocycler, Biometra GmbH, Göttingen Germany). Finally, PCR products were separated by horizontal electrophoresis (80 V, 400 mA, 240 min; Mini-Sub Cell GT Systems, Bio-Rad, Hercules, CA, United States) on a 5% agarose gel (Norgen Biotek Corp., Canada) [4].

**Table 1:** The new primer set flanking the (TTTAT)_3-5_ microsatellite located within the intron 6 of the LDHA gene.

Primer sequences	Microsatellite genotypes (bp)	Accession Nos.
F 5’-GAACACTGGAAGGAGGTCCA-3’ R 5’-TTCATCATTTGTTCTTGTGTTGC-3’	*3/3* ^*^ *3/4* *3/5* *4/4* *4/5* *5/5*	NW_004973198.1 AB744076.1 AB744077.1 AB744078.1

*number of repeat units of all of the potential genotypes.

### Urea-PAGE electrophoresis

After PCR amplification, samples were loaded onto a denaturing urea 20% polyacrylamide gels and electrophoretically separated for one hour at 20 W using Mini-PROTEAN Tetra cell (Bio-Rad, Hercules, CA, USA) electrophoretic chamber. Then, the gel was washed with 1 x TBE (ChemLand, Stargard Szczecinski, Poland) for about 15 min to remove the urea and soaked in 0.5 μg/ml ethidium bromide (AppliChem GmbH, Darmstadt, Germany) in aqueous solution for 45 min. The PCR products were examined under the UV light using UV*-*Transilluminator (Vilber Lourmat, Marne-la-Vallee Cedex, France) and each band of heterozygotes samples was isolated separately from the gel. DNA from desired amplicons were isolated by crush-and-soak method as was described elsewhere [36,37]. Concentration of each DNA sample was determined using the Quant-iT^TM^ dsDNA BR Assay Kit and a Qubit fluorometer (Invitrogen, Carlsbad, CA, USA). Afterwards, DNA samples were concentrated using Clean-Up Concentrator Kit (A&A Biotechnology, Gdańsk, Poland) following the vendor’s instructions and delivered to a custom DNA sequencing service.

Moreover, due to heteroduplexes DNA (in the course of PCR) urea-PAGE was conducted under identical experimental conditions as described above. Resulted gel was washed with 10% ethanol solution (Chempur, Piekary Slaskie, Poland) for 15 minutes and subsequently soaked in 1% nitric acid solution (ChemLand, Stargard Szczecinski, Poland) for 10 minutes. After washing step, gel was soaked in 0.2% silver nitrate solution with 0.075% formaldehyde solution (ChemLand, Stargard Szczecinski, Poland) for 30 minutes. Next gel was washed in 10% acetic acid solution (Chempur, Piekary Slaskie, Poland) for 10 minutes and subsequently washed twice in miliQ water. Immediately after staining gel image acquisition was performed using a GS-800™ Calibrated Densitometer (Bio-Rad, Hercules, CA, USA) with the aid of Quantity One^®^ 1-D image analysis software (Bio-Rad, Hercules, CA, USA).

### qPCR-HRM analysis

Individuals (genotyped using conventional PCR) were classified into five groups based on genotype type (3/3, 4/4, 3/4, 4/5, 5/5) and the results were tested by qPCR-HRM. PCR master mixtures contained 100 ng of genomic DNA (all gDNA samples were quantified using The Quant-iT^TM^ dsDNA Assay Kit; Life Technologies, Carlsbad, CA, USA), 250 nM of each primer, 4 μl of 5x AmpliQ HOT EvaGreen HRM Mix (Novazym, Poznań, Poland) and nuclease-free water (AppliChem GmbH, Darmstadt, Germany) in a total volume of 20 μl. The qPCR-HRM analysis was performed a Rotor-Gene Q instrument (QIAGEN GmbH, Hilden, Germany). The qPCR-HRM thermal profile was as follows: 15 min at 95°C for initial denaturation, subsequent 40 cycles (denaturation at 95°C for 15 s, primer annealing at 61°C for 20 s and product synthesis at 72°C for 20 s) and final elongation at 72°C for 1 min for complete product extension. Final elongation was then followed by pre-hold temperatures − 95°C for 15 s and 50°C for 15 s for product re-association and heteroduplex formation. Fluorescence data acquisition (melting curves) was performed during the melting phase (temperature change from 70 to 95°C at a transition rate of 0.1°C each step). High resolution melting curve analysis was performed using the Rotor-Gene Q Series Software 2.3.1 (QIAGEN GmbH, Hilden, Germany).

### Verification by DNA sequencing

PCR amplicons representing all identified genotypes (3/3, 4/4, 3/4, 4/5, 5/5) and additional amplicons (results obtained for (TTTAT)_3_/(TTTAT)_4_ and (TTTAT)_4_/(TTTAT)_5_ heterozygotes) isolated from urea-PAA gels were sequenced at the forward and reverse direction by each respective primer (**Table 1**), ABI Prism^™^ BigDye^™^ Terminator Cycle Sequencing Kit and ABI Prism^™^ Sequencer (Thermo Fisher Scientific, Waltham, Massachusetts, USA) by a custom DNA sequencing service (Genomed S.A., Warsaw, Poland). Three independent experiments were conducted to confirm the results obtained by sequencing. Sequences of all five samples were analysed using the BioEdit Sequence Alignment Editor (https://bioedit.software.informer.com/7.2/).

## Results and discussion

**Fig 1:**
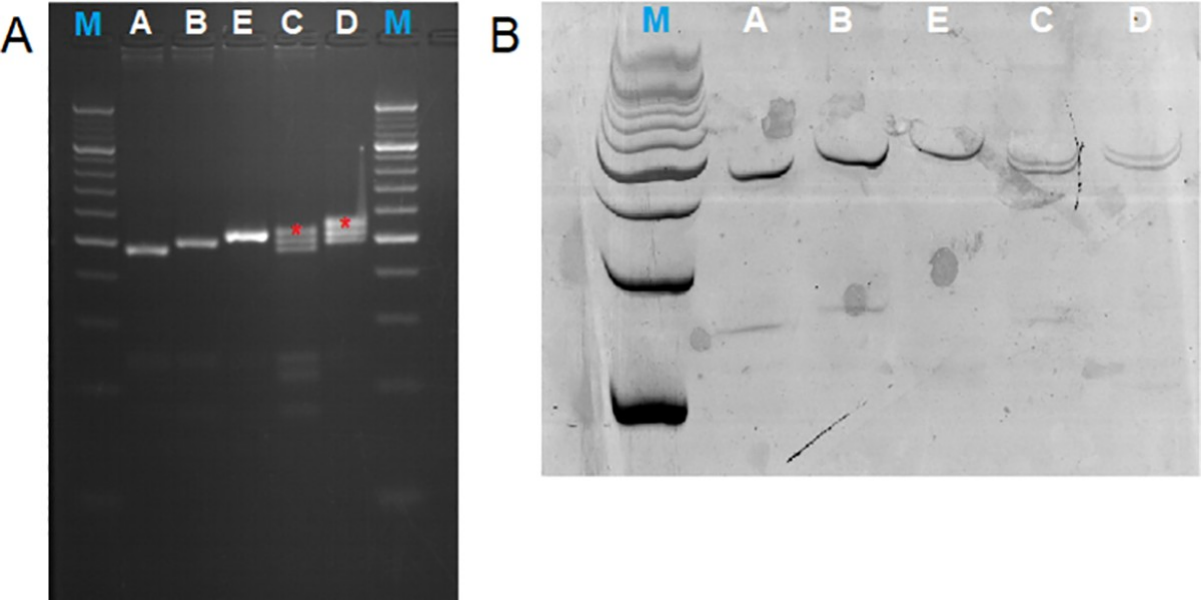
PCR analyses of five identified genotypes (A-E) using agarose gel electrophoresis (A) and urea-PAGE (B). Lines: M. O’RangeRuler 20 bp DNA Ladder (Thermo Scientific). A. (TTTAT)_3_/(TTTAT)_3_ homozygote. B. (TTTAT)_4_/(TTTAT)_4_ homozygote. C. (TTTAT)_3_/(TTTAT)_4_ heterozygote. D. (TTTAT)_4_/(TTTAT)_5_ heterozygote. E. (TTTAT)_5_/(TTTAT)_5_ homozygote. PCR plausible heteroduplexes marked by stars.

After PCR reaction, the obtained amplicons spanning the (TTTAT)_3-5_ microsatellite polymorphism within intron 6 of the *LDHA* gene were separated on a high-resolution agarose gel and the presence of five genotypes (of six potential) was confirmed in the analysed population of homing pigeons (**Fig 1A and 1B)**. Three homozygous genotypes (3/3, 4/4, 5/5) were visualised in agarose electrophoresis as a single amplicons. In contrast, two heterozygous genotypes 3/4, 4/5 (genotype 3/5 was affirmed of be absent in the examined individuals of homing pigeons) identified in agarose electrophoresis demonstrated two amplicons corresponded to alleles of different length and additional amplicon (heteroduplex) above PCR product (**Fig 1A**–heterozygote samples–C and D, additional amplicons–heteroduplexes were marked by stars). Due to additional band in heterozygous samples additional analysis was conducted to eliminate doubts about actual length of obtained PCR products. Moreover, chosen urea-PAGE method gave possibility to confirm our suspicious about the origin of unwanted amplicons in heterozygous samples. Result of electrophoresis under denaturing condition clearly confirmed five genotypes with two heterozygotes (**Fig 1B**).

**Fig 2:**
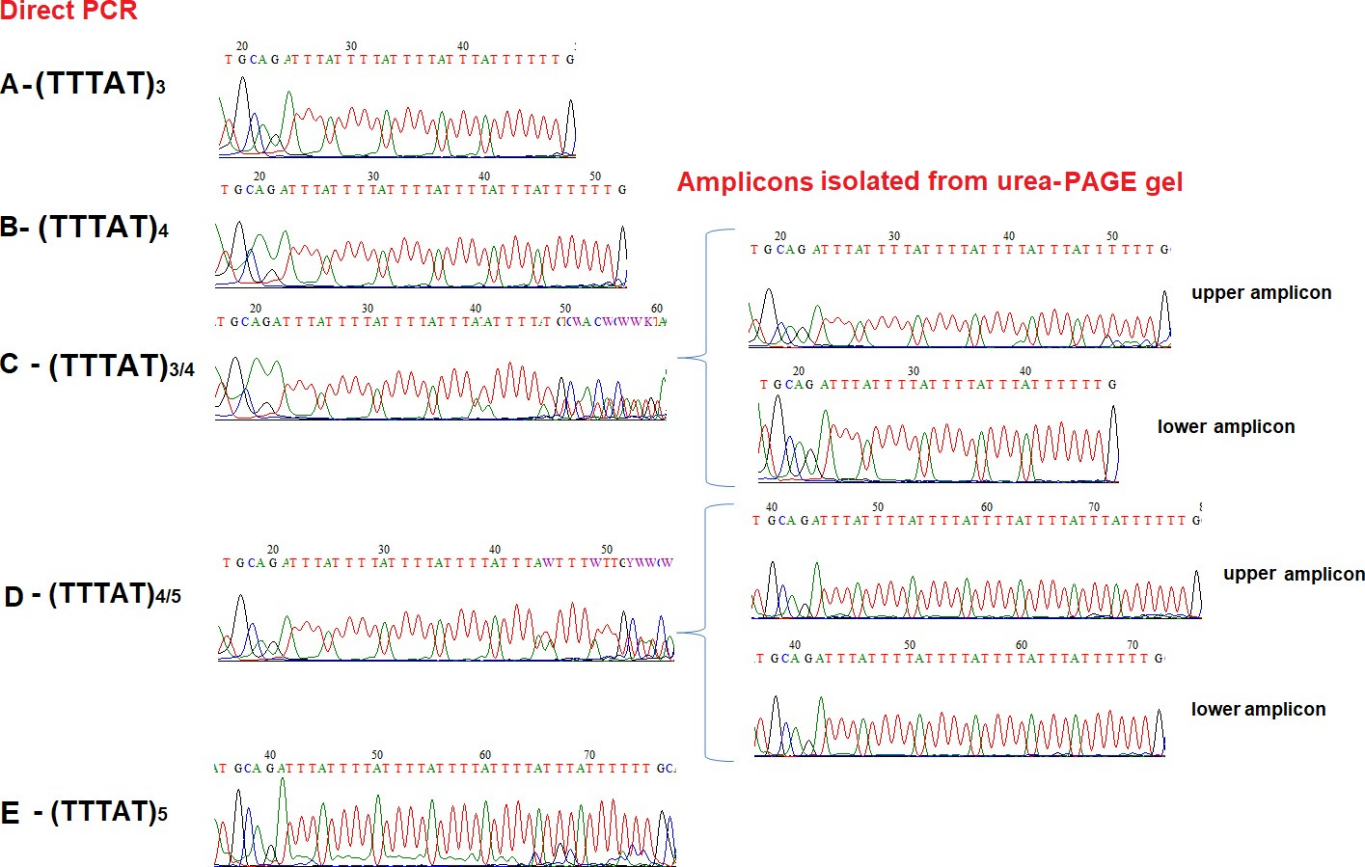
The sequencing results for the (TTTAT)_3-5_ microsatellite polymorphism in the *LDHA* gene. **A.** (TTTAT)_3_/(TTTAT)_3_ homozygote. **B.** (TTTAT)_4_/(TTTAT)_4_ homozygote. **C.** (TTTAT)_3_/(TTTAT)_4_ heterozygote. **D.** (TTTAT)_4_/(TTTAT)_5_ heterozygote. **E.** (TTTAT)_5_/(TTTAT)_5_ homozygote.

Simultaneously, sequencing method as a gold standard was performed to validate the results obtained by PCR. Three samples from each group (five groups, A-E) were arbitrarily selected from the whole analysed group and subjected to DNA sequencing. The results of sequencing were presented in **Fig 2**. The sequencing analyses were performed for each PCR product (left panel of **Fig 2**) and for each amplicon isolated out for polyacrylamide gels (right panel of **Fig 2**). Clearly, such proceedings demonstrated DNA sequence variation in the number of TTTAT repeat motifs. Based on sequencing, it was stated that samples A, B and E were homozygotes and contained (TTTAT)_3_, (TTTAT)_4_ and (TTTAT)_5_ repeat units, respectively. The two remaining samples, C and D, were identified as (TTTAT)_3_/(TTTAT)_4_ and (TTTAT)_4_/(TTTAT)_5_ heterozygotes, respectively. The most importantly the length of alleles (number of TTTAT repeat motif) of C and D samples were verified.

**Fig 3:**
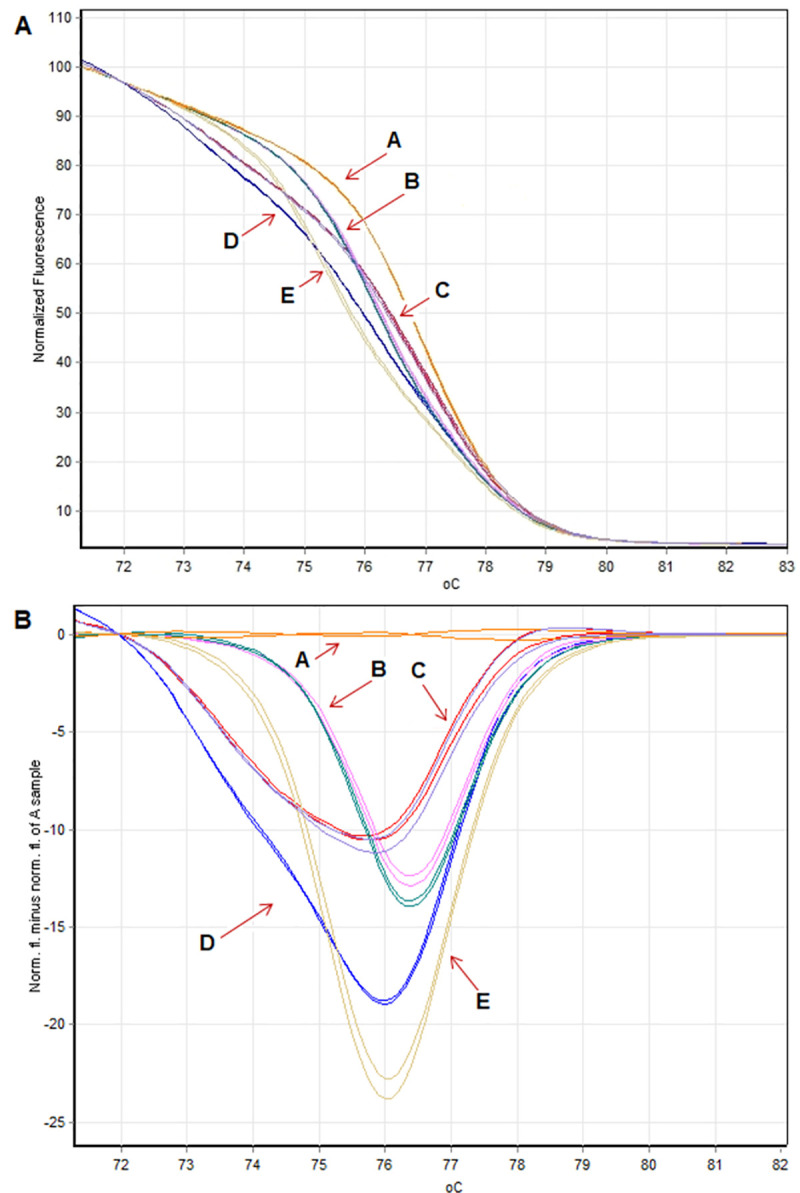
Results of qPCR-HRM analysis. **A.** The normalized HRM profile of five identified genotypes. **B.** The difference graph (sample A genotype 3/3 was selected as reference). A. (TTTAT)_3_/(TTTAT)_3_ homozygote. B. (TTTAT)_4_/(TTTAT)_4_ homozygote. C. (TTTAT)_3_/(TTTAT)_4_ heterozygote. D. (TTTAT)_4_/(TTTAT)_5_ heterozygote. E. (TTTAT)_5_/(TTTAT)_5_ homozygote.

Finally, preliminary results obtained using conventional PCR and sequencing were tested using the qPCR-HRM method. Based on melting curve analysis (MCA) five different melting profiles were recognized as a result of disassociation (melting) behaviour of each sample (**Fig 3A** and **3B**). The normalized HRM profiles and the difference graph (sample A genotype 3/3 was selected as reference) clearly show the five different melting profiles (A to E, **Fig 3**). HRM results showed no discrepancy between the identified alleles/genotypes from PCR and qPCR-HRM techniques.

Results obtained in conventional PCR and agarose electrophoresis analyses (**Fig 1A**) literally presented their limitations. Result of homozygote samples can be easily read, but in case of heterozygote samples the interpretation is limited due to additional amplicons (additional amplicons were marked with the asterisk; **Fig 1A**). Because the additional amplicons were observed only in heterozygotes we suggested that those amplicons are heteroduplexes. The heteroduplex is a double stranded DNA molecule that is formed by the annealing of two individual single-stranded DNA (derived from different alleles) of different repeat size during PCR. Presented findings correspond to Kulibaba and Liashenko [35] results. The appearance of additional fragments—heteroduplexes in heterozygous samples are common. The reason for heteroduplexes formation is presence of different in length alleles that exhibit different electrophoretic mobilities, that is why those structure are formed only in the case of heterozygotes [35]. Worth mentioning is fact that demonstrated artifacts are often situated near the target ones and cause difficulties in performing the precise genotyping [35], thus the main problem in this type of research is the specificity of the described polymorphism.

In our study solution of the problem described above was the application of the urea-PAGE to verify results observed in conventional PCR and agarose gel electrophoresis. As was presented in **Fig 1B** only two amplicons of different sizes were observed in denaturing PAGE, thus showing that the additional amplicons found in sample D and E in the agarose gel electrophoresis, are heteroduplexes. Moreover, precise determination of product lengths and in the number of TTTAT repeat units was obtained by DNA sequencing. Using the traditional Sanger sequencing method as gold standard in determination of analysed sequences. The microsatellite polymorphism can be also analysed using the traditional Sanger sequencing method, but microsatellite polymorphism analysis is challenging even for this technique. In our study sequencing of whole PCR product of heterozygotes did not solve the problem (left panel of **Fig 2**). In this type of microsatellite polymorphism it is necessary to analyse single amplicons isolated from the gel. Use of the agarose gel in this case did not provide sufficient resolution, especially in heterozygote samples, thus polyacrylamide electrophoresis was performed. This fact caused implementation of extensive laboratory procedures, low effectiveness, time-consumption methods and higher costs [38–40].

In order to avoid the limitations found in this study the HRM method was used for microsatellite polymorphism genotyping in the *LDHA* gene (**Fig 3**). Although the HRM is a post-PCR analysis (the melting pattern of the amplicons after products generation), in this study each sample represents one of five genotype. The interpretation of the results obtained via HRM is much easier than in conventional PCR and agarose electrophoresis analyses. Moreover, HRM method is much more efficient and less time-consuming that conventional methods. Hence, an accurate, powerful tool for genotype discrimination/genetic screening is needed. The high resolution melting analysis (HRM) has become an attractive and alternative method for determining different various genetic variations such as SNPs, insertions/deletions, microsatellite markers, DNA methylation, but also unknown DNA polymorphisms. HRM analysis is a one-tube and post-PCR method based on the intercalation of fluorescence dye into double-strand DNA. Fluorescence changes during dsDNA melting enable to discriminate genotypes [22]. High-resolution melting analysis is a relatively easy and rapid (the entire procedure can be completed in 1.5–2 hours), one-step, cost-effective application [22,41,42]. Moreover, it was found that STRs analysed by HRM had more genotypes than in capillary electrophoresis [22,41,42]. Demonstrated in this study method gave possibility to rapid discrimination of the (TTTAT)_3-5_ microsatellite polymorphism in the pigeon’s *LDHA* gene, that may serve as an rapid test for the commercial genotyping services. We suspect that microsatellite nature of analysed polymorphism in the *LDHA* gene caused lack of results in different populations of pigeons. Only Japanese and Egyptian population have been studied by Ramadan and colleagues [3,7] who presented four different genotypes (3/3, 4/4, 3/4, 4/5 repeats units for Japanese and 3/3, 4/4, 3/4, 5/5 for Egyptian birds) in the group of homing pigeons. Presented alternative approach–HRM analysis can potentially contribute to filling the knowledge gap about the frequency of *LDHA* microsatellite polymorphisms in pigeon populations and their potential effect on phenotypes.

## Conclusions

Herein, the (TTTAT)_3-5_ microsatellite polymorphism within intron 6 of the *LDHA* gene was analysed using the HRM method. The usability of HRM analysis of the selected microsatellite polymorphism was confirmed and the problem of the artifacts–heteroduplexes in heterozygote samples, which appear in the conventional amplification process was solved. The evaluated method can potentially contribute to filling the knowledge gap about the frequency of *LDHA* microsatellite polymorphisms in pigeon populations and their potential effect on phenotypes. Finally, provides a possibility for researches and breeders to see the complete picture of the significance of the *LDHA* gene in homing as well as non-homing pigeons.

## Supporting information

S1 Fig The original gel image of PCR analyses of five identified genotypes using agarose gel electrophoresis. From the left: O’RangeRuler 20 bp DNA Ladder (Thermo Scientific); (TTTAT)_3_/(TTTAT)_3_ homozygote; (TTTAT)_4_/(TTTAT)_4_ homozygote; (TTTAT)_3_/(TTTAT)_4_ heterozygote; (TTTAT)_4_/(TTTAT)_5_ heterozygote; (TTTAT)_5_/(TTTAT)_5_ homozygote; O’RangeRuler 20 bp DNA Ladder (Thermo Scientific), respectively.
(TIF)

S2 Fig The original urea-polyacrylamide gel electrophoresis of PCR analyses of five identified genotypes using agarose gel electrophoresis. From the left: O’RangeRuler 20 bp DNA Ladder (Thermo Scientific); (TTTAT)_3_/(TTTAT)_3_ homozygote; (TTTAT)_4_/(TTTAT)_4_ homozygote; (TTTAT)_3_/(TTTAT)_4_ heterozygote; (TTTAT)_4_/(TTTAT)_5_ heterozygote; (TTTAT)_5_/(TTTAT)_5_ homozygote, respectively.
(TIF)

S3 Fig The original image of qPCR-HRM analysis. The normalized HRM profile of five identified genotypes.
(TIF)

S4 Fig The original image of qPCR-HRM analysis. The difference graph (sample A genotype 3/3 was selected as reference).
(TIF)

S5 Fig The original image of sequencing results for the (TTTAT)_3-5_ microsatellite polymorphism in the *LDHA* gene (direct PCR). (TIF)

S6 Fig The original image of sequencing results for the (TTTAT)_3-5_ microsatellite polymorphism in the *LDHA* gene (amplicons isolated from urea-PAGE gel). (TIF)
