# Supplementary figures and images for: Application of PCR-HRM method for microsatellite polymorphism genotyping in the LDHA gene of pigeons (Columba livia)
Source: PLoS One. 2021 Aug 19;16(8):e0256065. doi: 10.1371/journal.pone.0256065 (PMC8376019; doi:10.1371/journal.pone.0256065)

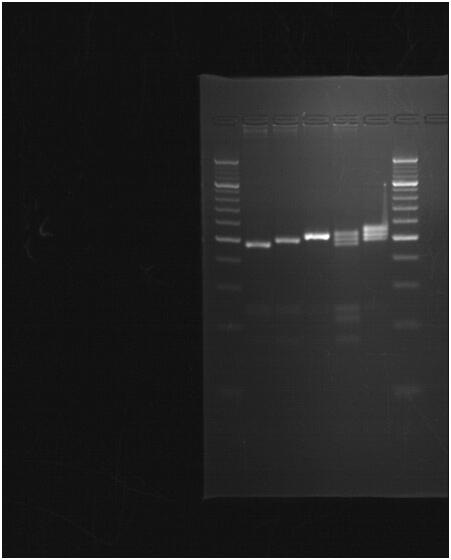

Supplement: S1 Fig — From the left: O’RangeRuler 20 bp DNA Ladder (Thermo Scientific); (TTTAT)3/(TTTAT)3 homozygote; (TTTAT)4/(TTTAT)4 homozygote; (TTTAT)3/(TTTAT)4 heterozygote; (TTTAT)4/(TTTAT)5 heterozygote; (TTTAT)5/(TTTAT)5 homozygote; O’RangeRuler 20 bp DNA Ladder (Thermo Scientific), respectively. (TIF) [file pone.0256065.s001.tif]

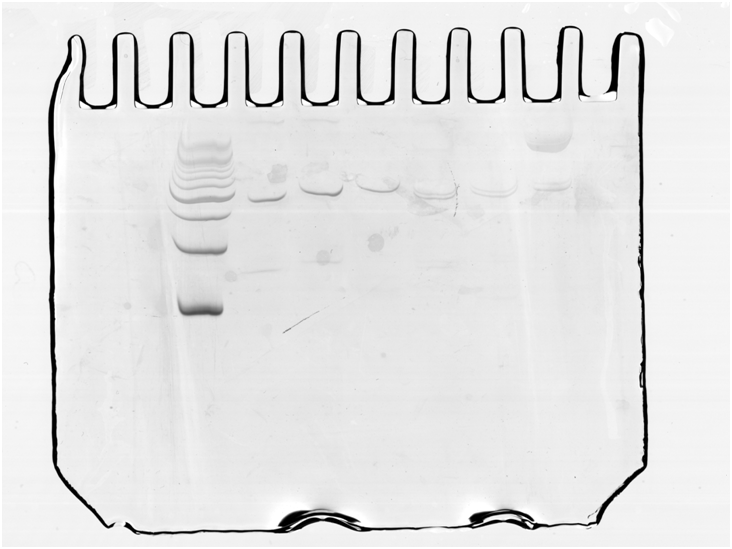

Supplement: S2 Fig — From the left: O’RangeRuler 20 bp DNA Ladder (Thermo Scientific); (TTTAT)3/(TTTAT)3 homozygote; (TTTAT)4/(TTTAT)4 homozygote; (TTTAT)3/(TTTAT)4 heterozygote; (TTTAT)4/(TTTAT)5 heterozygote; (TTTAT)5/(TTTAT)5 homozygote, respectively. (TIF) [file pone.0256065.s002.tif]

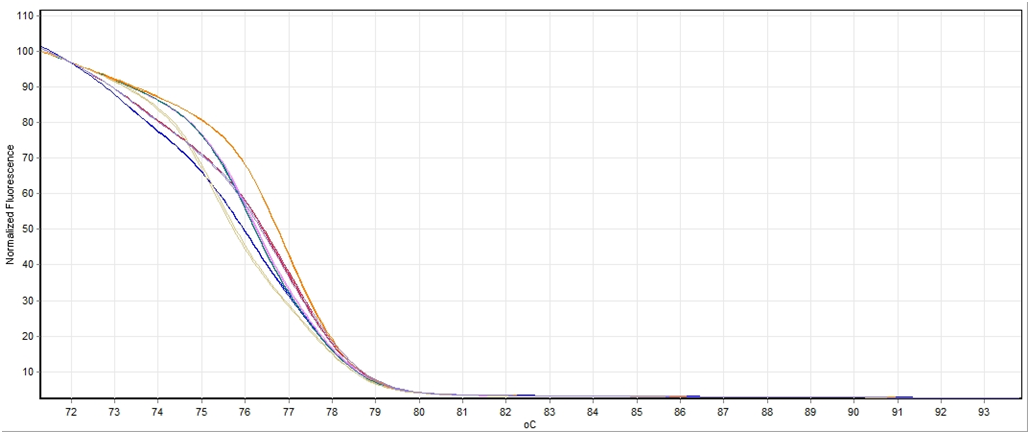

Supplement: S3 Fig — The normalized HRM profile of five identified genotypes. (TIF) [file pone.0256065.s003.tif]

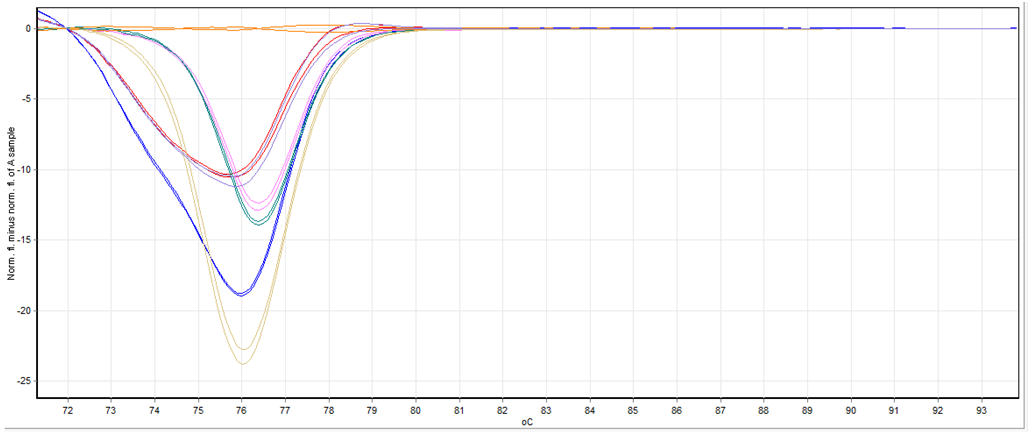

Supplement: S4 Fig — The difference graph (sample A genotype 3/3 was selected as reference). (TIF) [file pone.0256065.s004.tif]

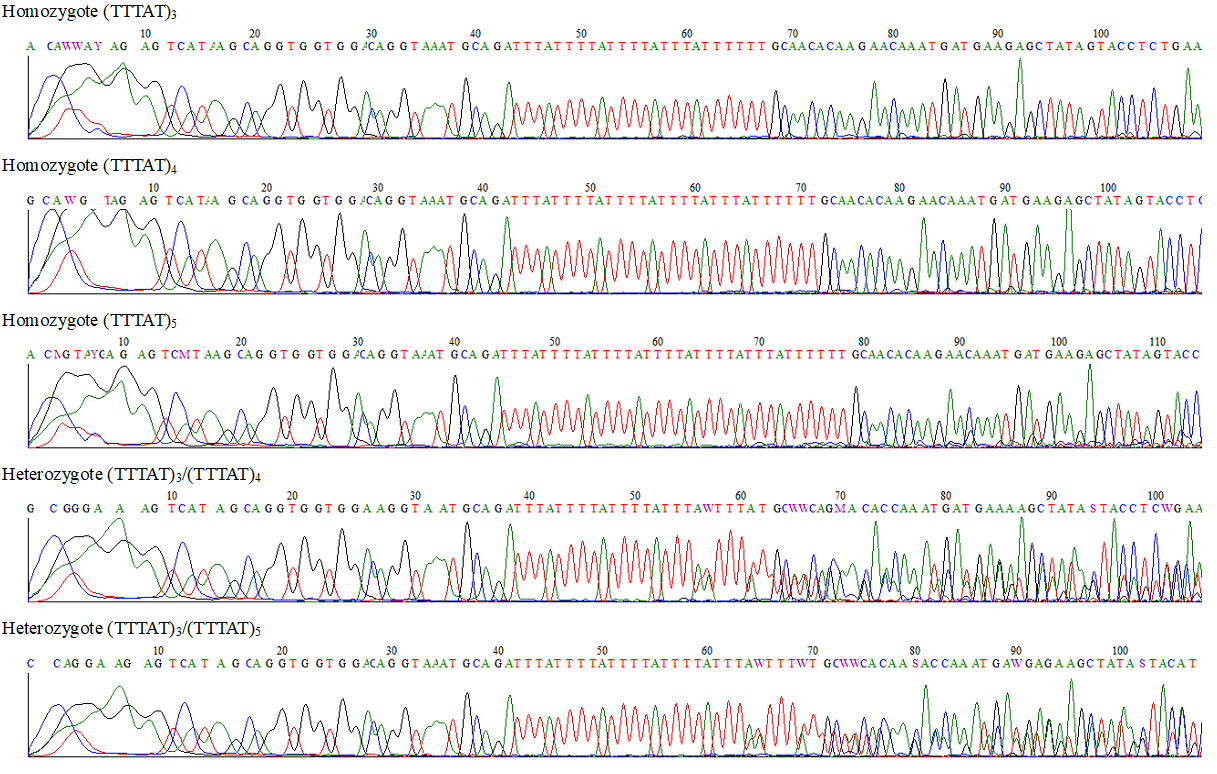

Supplement: S5 Fig — (TIF) [file pone.0256065.s005.tif]

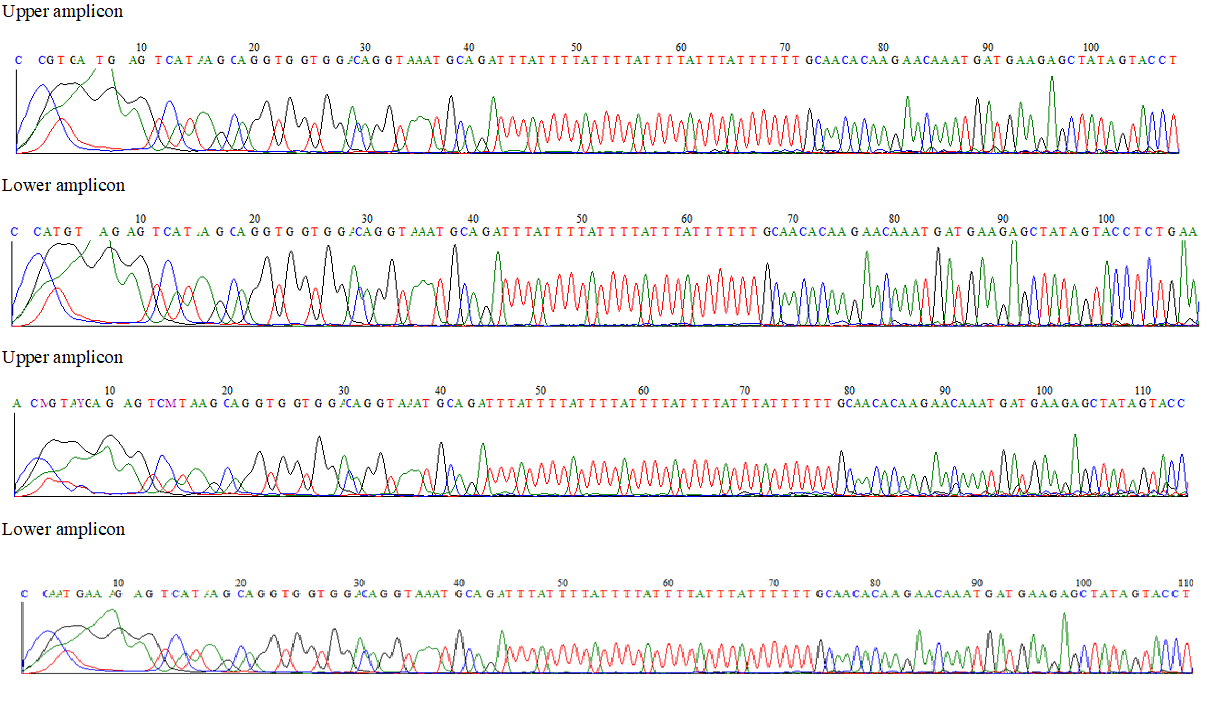

Supplement: S6 Fig — (TIF) [file pone.0256065.s006.tif]
